# Pulmonary redox imbalance drives early fibroproliferative response in moderate/severe coronavirus disease-19 acute respiratory distress syndrome and impacts long-term lung abnormalities

**DOI:** 10.1186/s13613-024-01293-3

**Published:** 2024-05-12

**Authors:** Chun Yang, Yuanyuan Tan, Zihao Li, Lei Hu, Yuanyuan Chen, Shouliang Zhu, Jiawei Hu, Tingting Huai, Mingqing Li, Guobin Zhang, Dewang Rao, Guanghe Fei, Min Shao, Zhenxing Ding

**Affiliations:** 1 The First Affiliated Hospital of Anhui Medical University, #218 Jixi Road, Hefei, 230022 Anhui China; 2 Anhui Medical University, #81 Meishan Road, Hefei, 230032 Anhui China

**Keywords:** COVID-19, Acute respiratory distress syndrome, Pulmonary fibrosis, Oxidative stress, Long COVID

## Abstract

**Background:**

COVID-19-associated pulmonary fibrosis remains frequent. This study aimed to investigate pulmonary redox balance in COVID-19 ARDS patients and possible relationship with pulmonary fibrosis and long-term lung abnormalities.

**Methods:**

Baseline data, chest CT fibrosis scores, N-terminal peptide of alveolar collagen III (NT-PCP-III), transforming growth factor (TGF)-β1, superoxide dismutase (SOD), reduced glutathione (GSH), oxidized glutathione (GSSG) and malondialdehyde (MDA) in bronchoalveolar lavage fluid (BALF) were first collected and compared between SARS-CoV-2 RNA positive patients with moderate to severe ARDS (n = 65, COVID-19 ARDS) and SARS-CoV-2 RNA negative non-ARDS patients requiring mechanical ventilation (n = 63, non-ARDS). Then, correlations between fibroproliferative (NT-PCP-III and TGF-β1) and redox markers were analyzed within COVID-19 ARDS group, and comparisons between survivor and non-survivor subgroups were performed. Finally, follow-up of COVID-19 ARDS survivors was performed to analyze the relationship between pulmonary abnormalities, fibroproliferative and redox markers 3 months after discharge.

**Results:**

Compared with non-ARDS group, COVID-19 ARDS group had significantly elevated chest CT fibrosis scores (p < 0.001) and NT-PCP-III (p < 0.001), TGF-β1 (p < 0.001), GSSG (p < 0.001), and MDA (p < 0.001) concentrations on admission, while decreased SOD (p < 0.001) and GSH (p < 0.001) levels were observed in BALF. Both NT-PCP-III and TGF-β1 in BALF from COVID-19 ARDS group were directly correlated with GSSG (p < 0.001) and MDA (p < 0.001) and were inversely correlated with SOD (p < 0.001) and GSH (p < 0.001). Within COVID-19 ARDS group, non-survivors (n = 28) showed significant pulmonary fibroproliferation (p < 0.001) with more severe redox imbalance (p < 0.001) than survivors (n = 37). Furthermore, according to data from COVID-19 ARDS survivor follow-up (n = 37), radiographic residual pulmonary fibrosis and lung function impairment improved 3 months after discharge compared with discharge (p < 0.001) and were associated with early pulmonary fibroproliferation and redox imbalance (p < 0.01).

**Conclusions:**

Pulmonary redox imbalance occurring early in COVID-19 ARDS patients drives fibroproliferative response and increases the risk of death. Long-term lung abnormalities post-COVID-19 are associated with early pulmonary fibroproliferation and redox imbalance.

**Graphical abstract:**

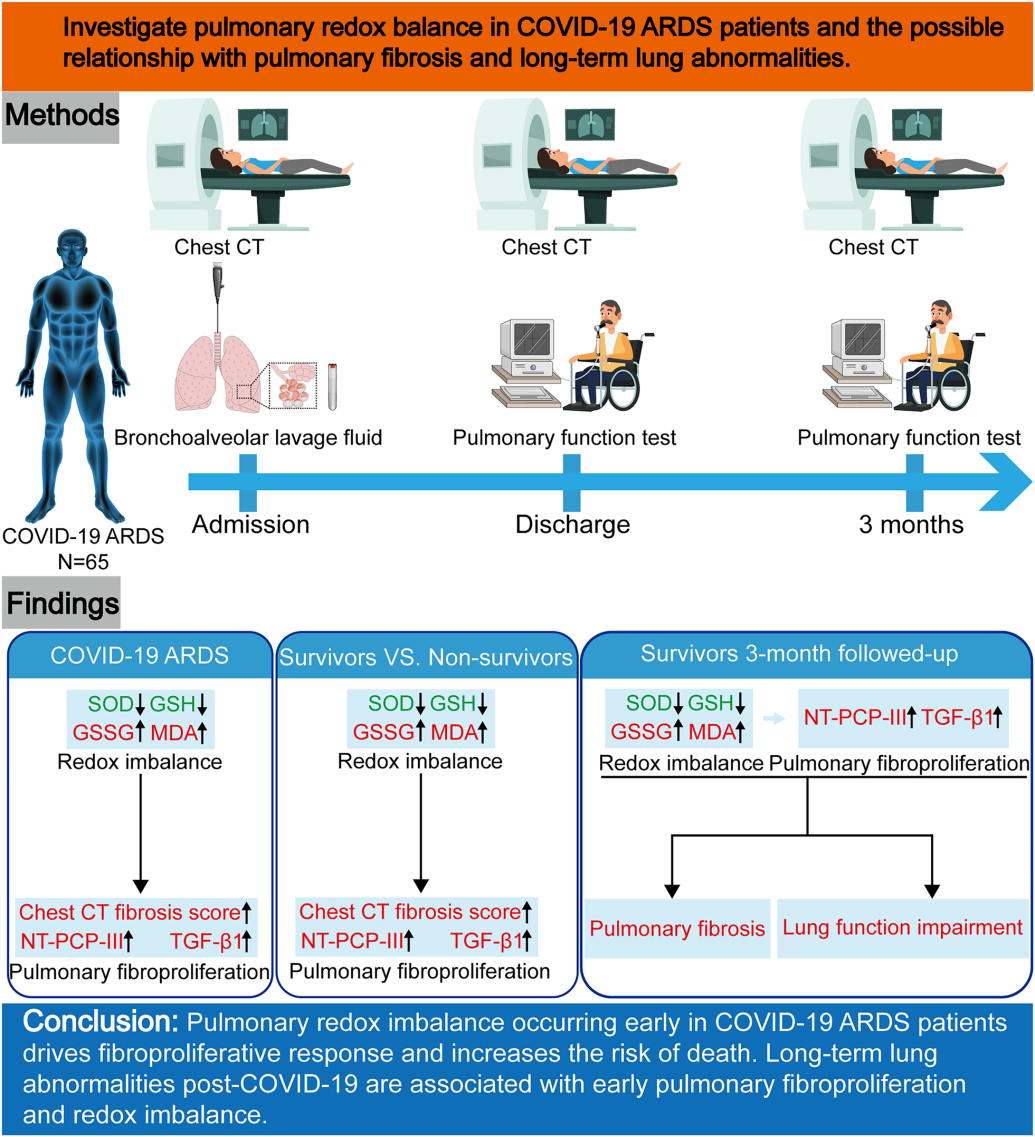

## Highlights

- Altering pulmonary redox balance drives an early pulmonary fibroproliferative response in COVID-19 ARDS.
- Pulmonary redox imbalance and fibroproliferation increase the risk of death in COVID-19 ARDS.
- Approximately 60% of moderate to severe COVID-19 ARDS survivors suffer residual pulmonary fibrosis and lung function impairment 3 months after hospital discharge.
- Pulmonary redox imbalance and fibroproliferation affect the resolution of residual pulmonary fibrosis and recovery from lung function impairment post-COVID-19.

## Introduction

Coronavirus disease 2019 (COVID-19) remains a pandemic, with infections and reinfections occurring worldwide [1]. Despite most patients suffering only mild symptoms, approximately 5–8% of COVID-19 patients evolve into acute respiratory distress syndrome (ARDS) [2, 3]. However, specific pharmacotherapy for COVID-19-associated ARDS has not been established, current treatments are limited to supportive care, and the mortality of COVID-19 ARDS remains at 30–40% [4, 5].

Given previous clinical evidence that ARDS-associated pulmonary fibrosis leads to prolonged mechanical ventilation, increased risk of death, and persistent lung abnormalities after discharge [6, 7]. The impact of severe COVID-19-associated pulmonary fibrosis on clinical outcomes and long-term prognosis is attracting the attention of researchers [8, 9]. Polak and colleagues systematically reviewed 42 reports on COVID-19 and found that COVID-19-associated pulmonary fibrosis occurred in 28 (22%) of a total of 129 patients [10]. Autopsy results from 26 critically COVID-19 patients with long disease duration also showed reduced severe acute respiratory syndrome coronavirus 2 (SARS-CoV-2) ribonucleic acid (RNA), decreased lung cell proliferation, and obvious pulmonary fibrosis [11]. Data from several recent follow-up studies have reported that radiographic residual pulmonary fibrosis, such as septal thickening, reticular changes, and traction bronchiectasis are still seen in 25% of COVID-19 ARDS survivors 3 months after discharge [12–14]. Moreover, impaired lung function persisted for one year or more after discharge in 30% to 60% of COVID-19 ARDS survivors, which correlated with residual pulmonary fibrosis on computed tomography (CT) [15–17]. These post-COVID-19 lung abnormalities often lead to dyspnea, cough, and fatigue, reducing quality of life and increasing the burden on society [18, 19].

The current views demonstrate that an aberrant wound-healing response stimulated by oxidative stress contributes to pulmonary fibrosis [20, 21]. During acute lung injury (ALI)/ARDS, viral RNA binds to toll-like receptors in alveolar epithelial cells (AECs) and macrophages, consequently up-regulating the expression of mitochondrial electron transport chain genes and the activation of nicotinamide adenine dinucleotide phosphate oxidase 2. This increases intracellular and extracellular production of reactive oxygen species (ROS) and reactive nitrogen species (RNS), which kills pathogens [22]. While under continuous viral infection and the storm of inflammatory factors, the reduction of antioxidant defense such as superoxide dismutase (SOD), reduced glutathione (GSH), accompanied by the accelerated production of ROS and RNS, led to the occurrence of redox imbalance [23]. Redox imbalance triggers oxidative stress and AECs suffer oxidative damage, causing an increase in the levels of oxidized glutathione (GSSG) and the lipid peroxidation product malondialdehyde (MDA) [24, 25]. Oxidative stress also induces AECs transdifferentiate to fibroblasts via epithelial-mesenchymal transition (EMT) [26, 27]. Furthermore, myofibroblasts under oxidative stress stimulation continue to activate and evolve apoptosis-resistant [28–30]. Unopposed secretion and deposition of extracellular matrix (ECM) by the fibroblasts and myofibroblasts leads to reduced lung compliance, hinders oxygen diffusion, and ultimately established pulmonary fibrosis [31, 32]. Although the exact molecular mechanisms of pro-fibrosis remain to be clarified in future research, oxidative stress is an important molecular mechanism in ARDS-associated pulmonary fibrosis [23, 33]. Available evidence suggests that SARS-CoV-2 infection of host cells involves redox imbalance and subsequent oxidative stress [34–37]. Through secondary analysis of publicly available COVID-19 transcriptome datasets, Narjes Saheb Sharif-Askari et al. revealed that oxidative stress genes are upregulated in lung tissue and the expression of these genes correlates with the severity of COVID-19 [38]. Previous lung biopsies have also demonstrated that SARS-CoV-2 infection inhibits nuclear factor (erythroid-derived 2)-like 2 (NRF2) gene expression, which suppresses NRF2-dependent antioxidant defenses [39]. These data provide evidence that pulmonary redox imbalance occurs in COVID-19. Although both in vivo and in vitro studies have suggested potential therapeutic benefits of antioxidants for pulmonary fibrosis after acute lung injury, translating these findings into clinical practice remains a challenge [40–42]. In contrast, there are rare clinical data on the relationship between pulmonary redox imbalance and ARDS-associated pulmonary fibrosis. Therefore, further research is needed to better elucidate the role of redox balance in pulmonary fibrosis and to identify factors that influence long-term lung abnormalities in survivors.

We hypothesized that redox imbalance drives early pulmonary fibroproliferation in COVID-19 ARDS and affects patient prognosis. To test the hypothesis, we prospectively collected chest CT and bronchoalveolar lavage fluid (BALF) from patients with moderate/severe COVID-19 ARDS. We then determined the concentrations of fibroproliferative biomarkers N-terminal peptide of alveolar collagen III (NT-PCP-III) and transforming growth factor-beta1 (TGF-β1). Antioxidant enzyme SOD concentration, redox state biomarkers GSH and GSSG levels, and the oxidative damage biomarkers MDA concentrations in BALF we also examined separately. We sequentially analyzed the association of redox balance with pulmonary fibrosis and mortality risk. Moreover, we followed moderate/severe COVID-19 ARDS survivors 3 months after discharge to assess the impact of redox balance on post-COVID-19 chest CT and lung function.

## Methods

### Study design and data collection

This prospective study was conducted from December 2022 to August 2023 in the intensive care unit and was approved by the Ethics Committee of the First Affiliated Hospital of Anhui Medical University (Ethics No.PJ20230745). Written informed consent was obtained from all enrolled patients. Consecutive patients were enrolled in the COVID-19 ARDS group when they fulfilled the following criteria: aged ≥ 18 years, intubated on admission and mechanically ventilated, confirmed SARS-CoV-2 infection from a pharyngeal swab sample at admission using real-time reverse transcriptase-polymerase chain reaction (RT-PCR) based tests, and had acute onset of moderate/severe ARDS, according to the Berlin criteria. Patients were excluded if they had active tumors, autoimmune diseases, were expected to die within the next 24 h, or refused consent. Patients enrolled in the non-ARDS group were those requiring mechanical ventilation for non-pulmonary respiratory failure (apoplexy, craniocerebral injury, central nervous system infection, poisoning) who did not meet the Berlin criteria for ARDS at any stage of their disease. Data related to demographics, comorbidities, steroid use, and peripheral blood test reports were collected in both groups. Among the comorbidities were chronic obstructive pulmonary disease, chronic heart failure, hypertension, diabetes mellitus, liver disease, and chronic kidney disease. Besides, days of mechanical ventilation and in-hospital mortality were recorded.

All diagnostic and therapeutic protocols were based on currently accepted ARDS guidelines and were supported by two specialists with more than 30 years of experience in the field, including but not limited to protopathy control, mechanical ventilation parameter settings, and fluid management. Patients in the COVID-19 ARDS group were classified as survivor subgroups if they improved and were discharged from the intensive care unit without mechanical ventilation. Survivors in the COVID-19 ARDS group were discharged with a CT scan of the chest and pulmonary function tests and were invited again to a dedicated outpatient follow-up 3 months after discharge. Research funding covered the cost of specific tests for patients enrolled during the study.

### Chest CT scan and pulmonary fibrosis scoring

All enrolled patients underwent chest high-resolution CT scans on admission using a SOMATOM Sensation 64 Multi-Slice CT Scanner (Siemens Healthineers, Germany). All chest CT scans were acquired at full inspiration from the lung apex to the base, and the CT scans were obtained with 1.25 mm thickness.

The method established by Ichikado et al. was used for describing the CT scan score of pulmonary fibrosis validated with a pathological examination [43, 44]. This scoring system categorizes chest CT manifestations of alveolar and interstitial abnormalities into levels 1 to 6 (Table 1). The presence of these six abnormalities was independently evaluated in three zones (above the tracheal carina, between the tracheal carina and left pulmonary vein, and below the left pulmonary vein) of each lung. The degree of each abnormality was measured by visually estimating the percentage (10% accuracy) of impaired lung parenchyma in each zone. The chest CT scans were analyzed blindly by two designed radiologists, who scored all scans independently. Another independent thoracic radiologist made the final decision when a difference of over 10% between the two designed radiologists. Two radiologists averaged the results of each area. The fibrosis score for each selected zone was computed by multiplying the area percentage by the point value. The six-zone scores were averaged to obtain the final score for each abnormality per patient. The total CT score for each enrolled patient was obtained by summing the six averaged scores.

**Table 1:** Chest CT pulmonary fibrosis scoring rules

Chest CT Signs	Point value
Normal attenuation	1
Ground-glass attenuation	2
Consolidation	3
Ground-glass attenuation with traction bronchiectasis	4
Consolidation with traction bronchiectasis	5
Honeycombing	6

### Bronchoalveolar lavage fluid collection

Fiberoptic bronchoscopy not only collected BALF but also aspirated sputum, assisted in the diagnosis of lung infections. BALF was obtained from the most infiltrated lung area on the chest radiograph and was performed by one of two assigned clinicians to ensure consistency of technique. Bronchoalveolar lavage was performed by dropping 10 ml of sterile saline into the lesion. If less than 10 ml of lavage fluid was recovered, a second 10 ml of sterile saline was administered. The collected BALF was centrifuged at 1200 × g for 10 min and the supernatant was frozen at −80 °C for subsequent batch analysis.

### Laboratory analyses

NT-PCP-III (Elabscience Biotechnology, China), TGF-β1 (Elabscience Biotechnology, China), SOD activity (COIBO Biotechnology, China), GSH (COIBO Biotechnology, China), GSSG (COIBO Biotechnology, China) and MDA (COIBO Biotechnology, China) levels in BALF were determined by double antibody-coated tube radioimmunoassay method, according to the instructions of the kit. Briefly, BALF was added to specific antibody-coated microtiter plate wells. Then horseradish peroxidase-labeled detection antibody was added and incubated to form antibody–antigen–enzyme–antibody complex. After washing, 3,3′,5,5′-tetramethylbenzidine solution was added to react with horseradish peroxidase for color development, and the optical density was measured at a wavelength of 450 nm with spectrophotometer. Finally, the concentration of biomarkers in the BALF was calculated by standard curve. The assay was repeated three times for each sample and the average of these three independent experiments was used as the final measured concentration.

### Sample size estimation and statistical analysis

Based on previous clinical data, the mortality rate for moderate-to-severe ARDS is about 40%, and the mortality rate for non-ARDS patients needing mechanical ventilation is about 18%. In this research, a statistical power of 0.8 and a probability of type I error of 0.05 were set, and the absolute difference in detectable mortality was finally calculated for 65 patients in each of the two groups.

For continuous data, the normality test was performed first, the mean ± standard deviation was used to describe data with a normal distribution, and the median (25th to 75th percentile) was used to describe data with a non-normal distribution. Student’s t-test was used for comparisons between groups when both sets of data were normally distributed with equal variance, otherwise Mann–Whitney U-test was used. For categorical data, the chi-squared test (Fisher’s exact test) was used to compare differences between groups. Spearman correlation was performed to analyze the relationship between chest CT fibrosis scores or fibroproliferative markers and pulmonary redox balance parameters. The two-tailed p-value ≤ 0.05 was statistically significant. All statistical analyses were performed using GraphPad Prism Software (version 9.5.0 for Windows, San Diego, USA).

## Results

### Baseline characteristics of enrolled patients

**Fig. 1:**
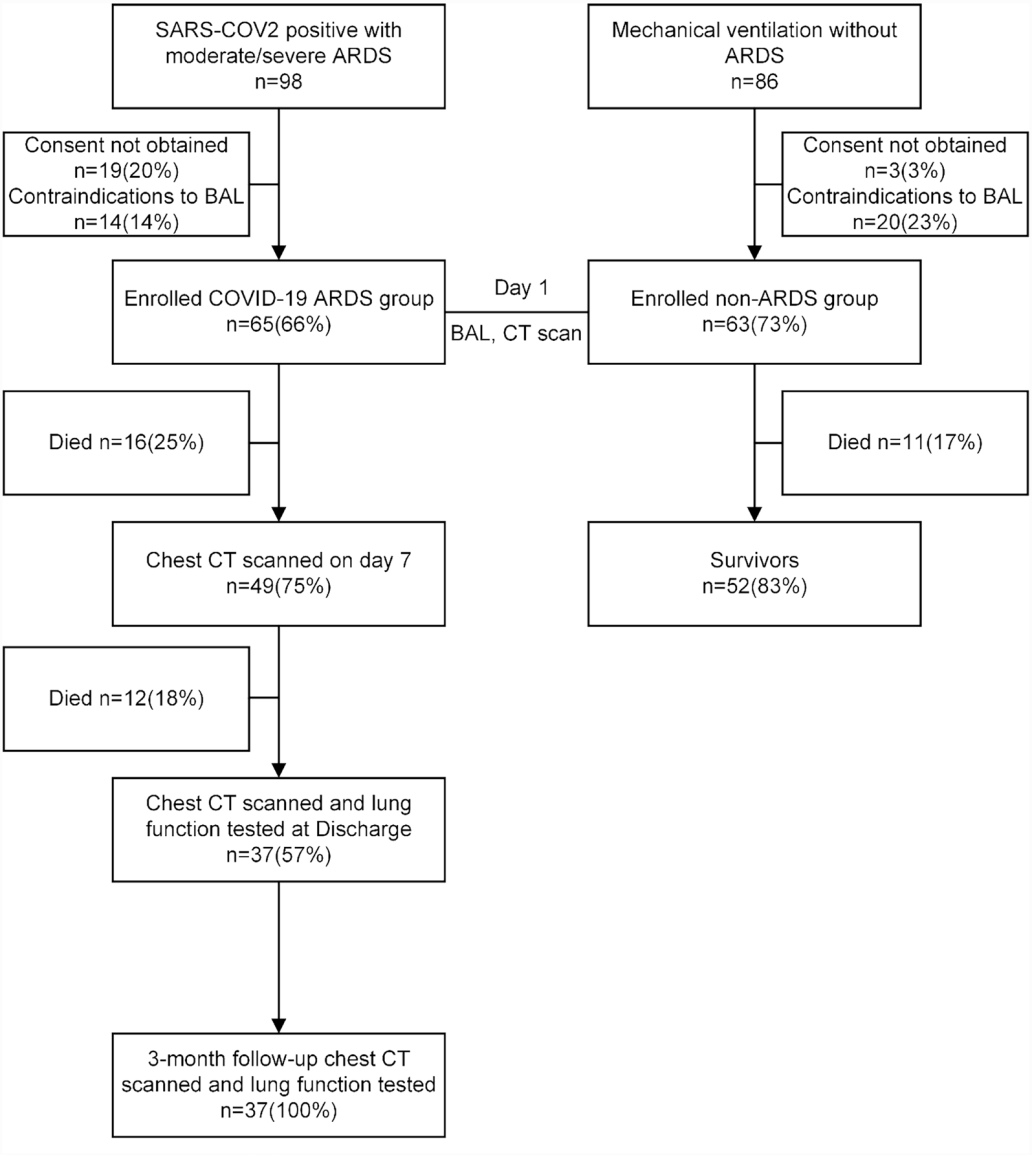
Flow chart of the clinical research protocol

Between December 2022 and August 2023, this prospective, single-center, cohort study evaluated 98 eligible patients who were admitted with moderate to severe ARDS for SARS-CoV-2 infection. Of these, 65 patients were enrolled in the study and assigned to the COVID-19 ARDS group (Fig. 1). Sixty-three control non-ARDS patients were recruited, 32 (51%) patients had apoplexy, 24 (38%) patients were admitted due to closed craniocerebral injury, 5 (8%) patients had central nervous system infection, and 2 (3%) patients with poisoning. No significant differences in age, sex ratio, BMI, clinical severity scores, smoking proportion, and associated comorbidities were observed between the two groups of patients (Table 2). However, COVID-19 ARDS patients showed a lower oxygenation index (p < 0.001), higher 30-day mortality (p = 0.002), and longer duration of mechanical ventilation (p = 0.015) compared to non-ARDS patients. In addition, although there was no difference in CRP (p = 0.084) between the two groups of patients on admission, ferritin (p < 0.001) and D-dimer (p < 0.001) levels were significantly elevated in COVID-19 ARDS patients compared to non-ARDS.

**Table 2:** Clinical Characteristics from COVID-19 ARDS and non-ARDS patients

Parameters	COVID-19 ARDS	Non-ARDS	p-Value
	n = 65	n = 63	
Age, years^a^	66 (58.5–73.5)	63 (55–74)	0.227
Sex, females(%)	34 (52.31%)	31 (49.21%)	0.860
BMI, kg/m^2^^b^	21.0 ± 2.79	21.32 ± 3.02	0.638
PaO_2_/FiO_2_^b^	123.5 ± 29.81	210.7 ± 35.90	**< 0.001**
APACHE-II score^b^	23.66 ± 3.86	23.16 ± 4	0.469
SOFA score^b^	6.95 ± 3.15	6.67 ± 2.92	0.594
Smoking, n(%)	10 (15.38%)	12 (22.64%)	0.349
Comorbidity, n(%)			
Chronic obstructive pulmonary disease	9 (13.85%)	6 (9.52%)	0.585
Chronic heart failure	22 (33.85%)	19 (30.16%)	0.707
Hypertension	58 (89.23%)	54 (85.71%)	0.601
Diabetes mellitus	19 (29.23%)	15 (23.81%)	0.551
Liver disease	6 (9.23%)	3 (4.76%)	0.492
Chronic kidney disease	27 (41.54%)	28 (44.44%)	0.858
CRP, mg/L^b^	152.3 ± 77.89	154.8 ± 70.5	0.847
Ferritin, ng/mL^b^	1382 ± 693.8	481.9 ± 298.2	**< 0.001**
D-dimer, ng/mL^b^	1332 ± 660	619.4 ± 360.8	**< 0.001**
Use of steroids for treatment, n(%)	25 (38.46%)	9 (14.29%)	**0.003**
Length of mechanical ventilation, days^a^	14 (10.5–18.5)	12 (8–16)	**0.015**
30-day Mortality, n(%)	28 (43.08%)	11 (17.46%)	**0.002**

Abbreviations: *ARDS* acute respiratory distress syndrome, *BMI* body mass index, *APACHE II* acute physiology and chronic health evaluation II, *PaO*_*2*_*/FiO*_*2*_ arterial oxygen tension pressure/fraction of inspired oxygen, *SOFA* sequential organ failure assessment, *CRP* C-reactive protein

Bold indicates a statistically significant difference

^a^Data are described as median values (25th to 75th percentile)

^b^Data are described as mean values ± standard deviation

### Pulmonary fibroproliferative response occurs early in COVID-19 ARDS patients

**Fig. 2:**
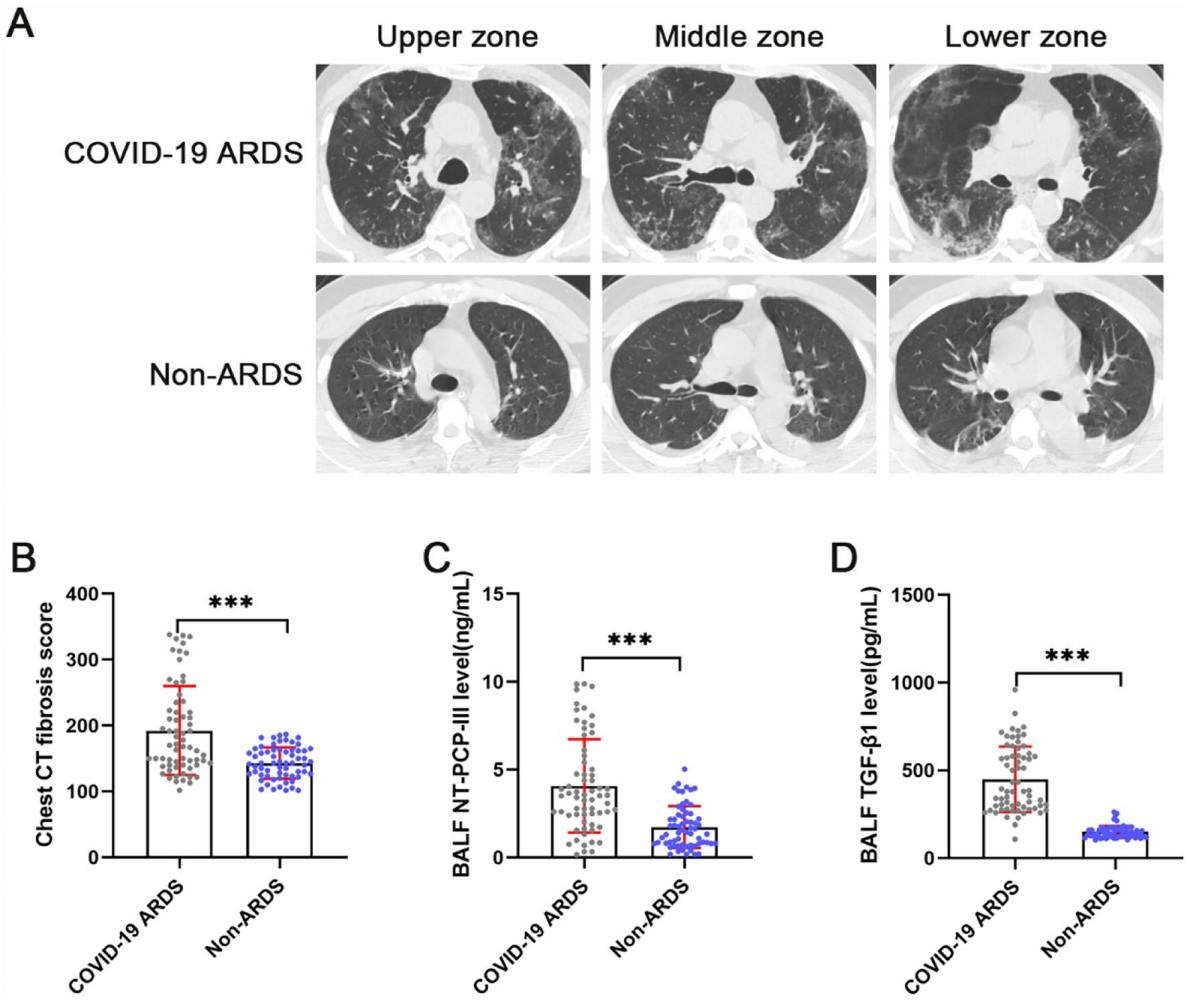
Pulmonary fibroproliferative response in COVID-19 ARDS patients on admission. **A** Chest CT scans on admission in COVID-19 ARDS and non-ARDS patients. **B, C, **and** D** Comparison of chest CT fibrosis score, NT-PCP-III, and TGF-β1 levels of bronchoalveolar lavage fluid in COVID-19 ARDS and non-ARDS patients. ^*^p < 0.05, ^**^p < 0.01, ^***^p < 0.001. NS, no statistically significant difference

As consent for this study was obtained from authorized relatives of the patients before patient enrollment, BAL and chest CT scans were performed on admission in both groups. None of the patients had emergencies during BAL and chest CT scans. As shown in Fig. 2A, COVID-19 ARDS patients exhibited bilateral scattered ground-glass attenuation on lung CT at admission, whereas patients with non-ARDS exhibited almost normal attenuation in both lungs or only ground-glass attenuation in the lobes. To compare the two groups of patients with early pulmonary fibroproliferation, chest CT fibrosis score was performed. The result was that the chest CT fibrosis score on admission was higher in the COVID-19 ARDS group (p < 0.001) than in the non-ARDS group (Fig. 2B). We further measured the fibroproliferative markers NT-PCP-III and TGF-β1 in both groups of patients. The concentrations of NT-PCP-III (p < 0.001) and TGF-β1 (p < 0.001) were significantly elevated in BALF from COVID-19 ARDS patients compared with non-ARDS patients (Fig. 2C, 2D). These results suggest that the pulmonary fibroproliferative response had already initiated in COVID-19 ARDS patients on admission.

### Pulmonary redox imbalance drives early fibroproliferation and increases the risk of death in COVID-19 ARDS patients

**Fig. 3:**
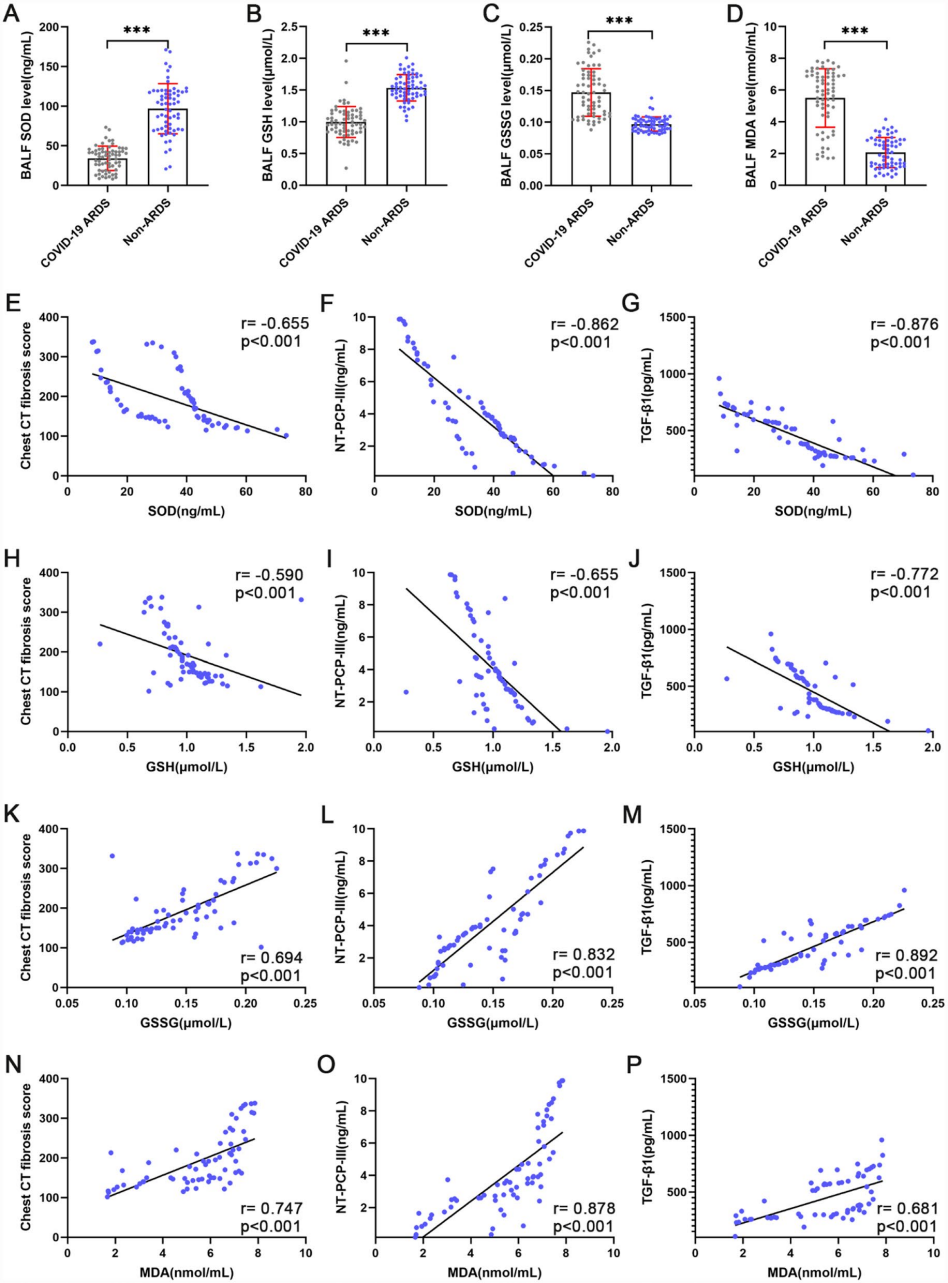
Pulmonary redox imbalance in COVID-19 ARDS patients. **A–D** Comparison of pulmonary antioxidant markers and markers of oxidative damage in COVID-19 ARDS and non-ARDS patients. **E–G** Spearman correlation analysis between SOD and chest CT fibrosis score (**E**), NT-PCP-III levels (**F**), TGF-β1 levels (**G**) of bronchoalveolar lavage fluid in COVID-19 ARDS patients. **H–J** Spearman correlation analysis between GSH and chest CT fibrosis score (**H**), NT-PCP-III levels (**I**), TGF-β1 levels (**J**) of bronchoalveolar lavage fluid in COVID-19 ARDS patients. **K–M** Spearman correlation analysis between GSSG and chest CT fibrosis score (**K**), NT-PCP-III levels (**L**), TGF-β1 levels (**M**) of bronchoalveolar lavage fluid in COVID-19 ARDS patients. **N–P** Spearman correlation analysis between MDA and chest CT fibrosis score (**N**), NT-PCP-III levels (**O**), TGF-β1 levels (**P**) of bronchoalveolar lavage fluid in COVID-19 ARDS patients. ^*^p < 0.05, ^**^p < 0.01, ^***^p < 0.001. NS, no statistically significant difference

We investigated the pulmonary redox balance of COVID-19 ARDS patients by measuring biomarkers of antioxidant defense, redox state, and oxidative damage in BALF. Pulmonary antioxidant defense was assessed by measuring the level of SOD. As shown in Fig. 3A, SOD levels in BALF were lower in the COVID-19 ARDS group than in the non-ARDS group (p < 0.001). To study the pulmonary redox status, GSH and GSSG levels were determined in BALF. We observed lower GSH (p < 0.001) levels and significantly higher GSSG (p < 0.001) levels in the BALF of the COVID-19 ARDS group compared to the non-ARDS group (Fig. 3B, C). Oxidative damage in the lung was evaluated by determining the MDA levels of BALF. Of interest, MDA concentrations in BALF were significantly elevated in COVID-19 ARDS patients (p < 0.001) compared to non-ARDS (Fig. 3D). The above results suggest that pulmonary redox balance is disrupted in COVID-19 ARDS patients, manifested as decreased levels of antioxidant enzymes in the lungs and increased oxidative damage.

To investigate possible relationships between early pulmonary fibroproliferation and pulmonary redox system status in COVID-19 ARDS patients, spearman correlation analysis was used between pulmonary redox balance marker measurements and pulmonary fibroproliferative indicators. Of note, the SOD and GSH levels in BALF were both inversely related to Chest CT fibrosis scores, as well as NT-PCP-III and TGF-β1 concentrations in BALF (Fig. 3E–J). On the contrary, the GSSG and MDA levels in BALF were both directly related to Chest CT fibrosis scores, as well as NT-PCP-III and TGF-β1 concentrations in BALF (Fig. 3K–P). These data indicate that early pulmonary fibroproliferation is a potential consequence of a pro-oxidative stress state resulted from pulmonary redox imbalance in COVID-19 ARDS.

**Fig. 4:**
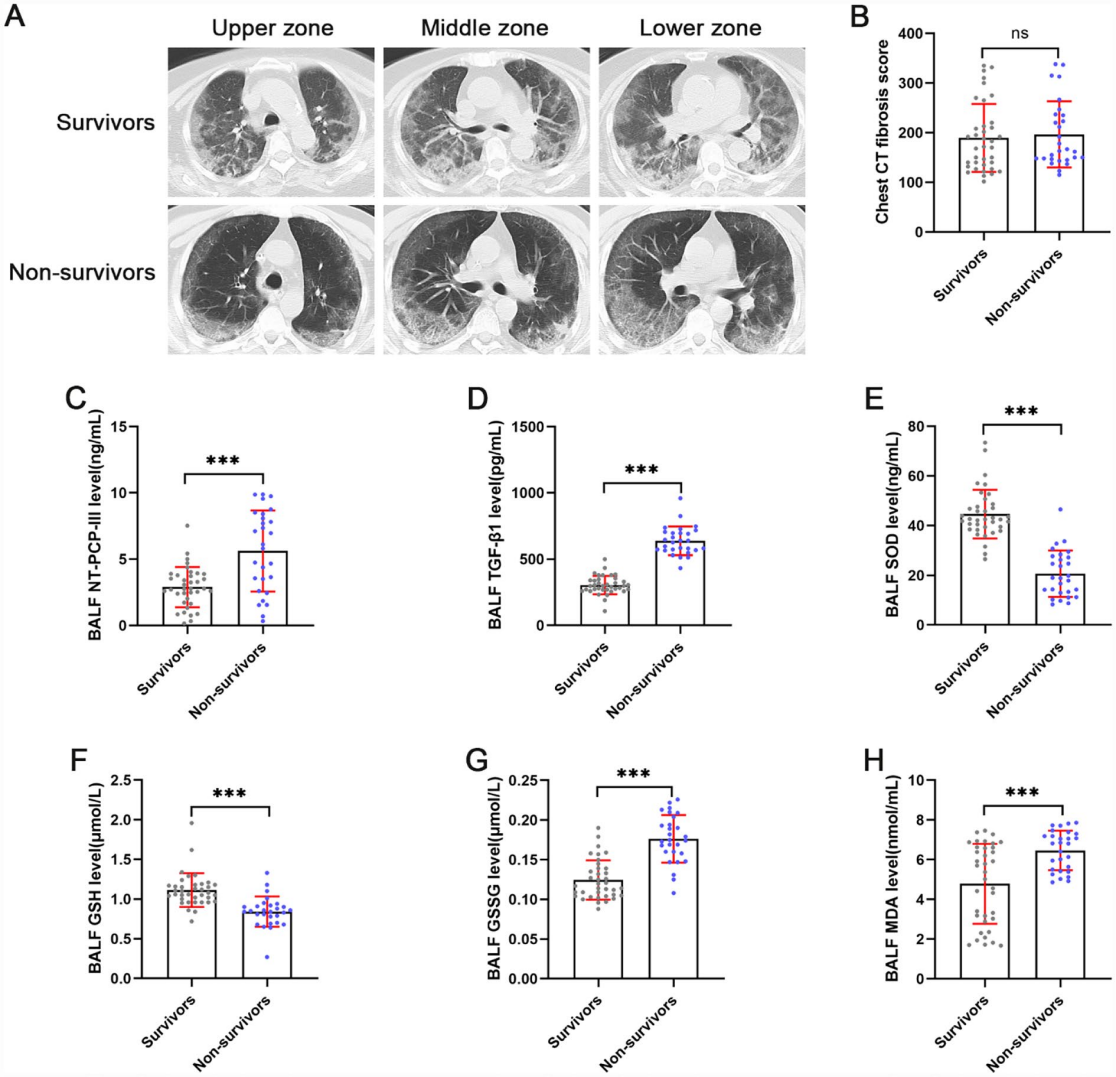
Impact of pulmonary redox imbalance on survival outcomes in COVID-19 ARDS patients. **A** Chest CT scans on admission in COVID-19 ARDS survivors and non-survivors. **B** Comparison of chest CT scores on admission between COVID-19 ARDS survivors (n = 37) and non-survivors (n = 28). (**C**–**H**) Comparison of NT-PCP-III, TGF-β1, SOD, GSH, GSSG, and MDA levels of bronchoalveolar lavage fluid in COVID-19 ARDS survivors (n = 37) and non-survivors (n = 28). ^*^p < 0.05, ^**^p < 0.01, ^***^p < 0.001. NS, no statistically significant difference

Subgroup analysis of survivors and non-survivors in the COVID-19 ARDS group was performed to assess the impact of pulmonary redox imbalance and concomitant fibroproliferation on survival outcomes. Survivors and non-survivors in the COVID-19 ARDS group had similar demographics at enrollment, while non-survivors severity scores were significantly higher than survivors (Table 3). Correspondingly, circulating ferritin (p = 0.002) and D-dimer (p = 0.049) levels were higher in non-survivors as compared to survivors. Figure 4A presents CT scan images of the chest at admission for survivors and nonsurvivors of the COVID-19 ARDS group, both subgroups showed bilateral scattered ground-glass attenuation. Despite no difference in chest CT fibrosis scores between the two subgroups of patients, fibroproliferative markers NT-PCP-III (p < 0.001) and TGF-β1 (p < 0.001) were significantly higher in non-survivors BALF than in survivors (Fig. 4B–D). In addition, our data also showed lower SOD (p < 0.001) and GSH (p < 0.001) levels, and significantly higher GSSG (p < 0.001) and MDA (p < 0.001) levels in non-survivors BALF compared to survivors (Fig. 4E–H).

**Table 3:** Clinical Characteristics from COVID-19 ARDS group survivors and non-survivors patients

Parameters	Survivors	Non-survivors	p-Value
	n = 37	n = 28	
Age, years^a^	66 (58.5–73.5)	66.5 (55.75–73.75)	0.790
Sex, females(%)	19 (51.35%)	16 (57.14%)	0.802
BMI, kg/m^2*^	21.31 ± 2.73	20.78 ± 2.89	0.448
PaO_2_/FiO_2_^b^	122.5 ± 24.1	124.9 ± 36.45	0.746
APACHE-II score^b^	22.68 ± 3.43	24.96 ± 4.07	**0.017**
SOFA score^b^	5.56 ± 2.36	8.75 ± 3.18	**< 0.001**
Smoking, n(%)	6 (16.22%)	4 (14.29%)	NS
Comorbidity, n(%)	25 (67.57%)	20 (71.43%)	0.792
CRP, mg/L^b^	153.2 ± 80.23	151.1 ± 76.12	0.914
Ferritin, ng/mL^b^	1155 ± 595.2	1681 ± 710.6	**0.002**
D-dimer, ng/mL^b^	1193 ± 640	1517 ± 651.4	**0.049**
Use of steroids for treatment, n(%)	12 (32.43%)	13 (46.43%)	0.307

Abbreviations: *ARDS* acute respiratory distress syndrome, *BMI* body mass index, *APACHE II* acute physiology and chronic health evaluation II, *PaO*_*2*_*/FiO*_*2*_ arterial oxygen tension pressure/fraction of inspired oxygen, oxygenation index, *SOFA* sequential organ failure assessment, *CRP* C-reactive protein

Bold indicates a statistically significant difference

^a^Data are described as median values (25th to 75th percentile)

^b^Data are described as mean values ± standard deviation

### Relationship between chest CT abnormalities, and lung function impairment at 3-month follow-up and pulmonary redox imbalance on admission

**Fig. 5:**
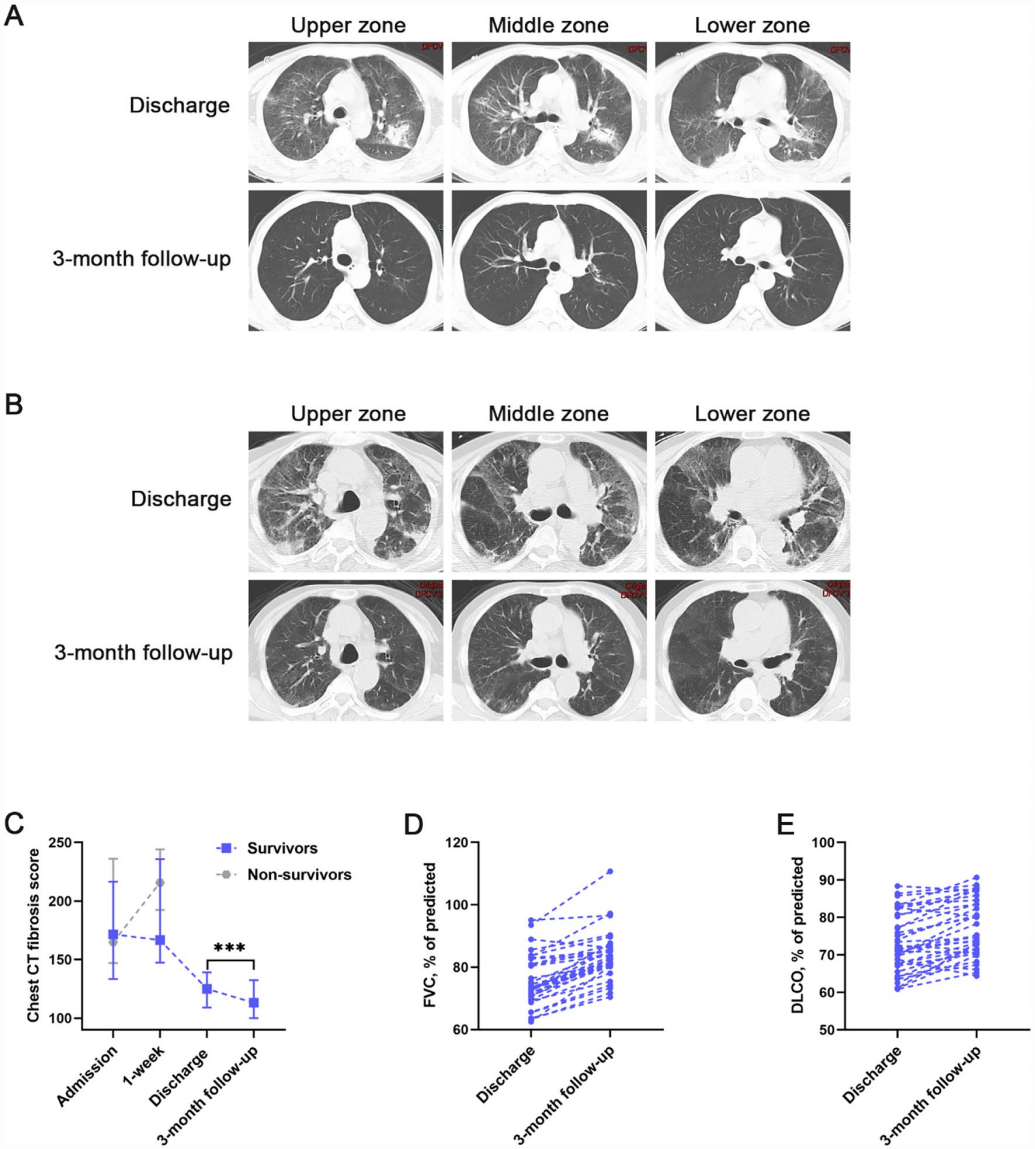
COVID-19 ARDS survivors 3-month followed-up with chest CT scan and pulmonary function tests. **A** CT scan images from a 66-year-old male COVID-19 ARDS survivor. On discharge, chest CT scan showed multiple ground-glass attenuation in both lungs, with consolidation and traction bronchiectasis; at 3-month follow-up, the chest CT scan showed ground-glass attenuation only in a few areas of the left lung. **B** CT scan images from a 63-year-old male COVID-19 ARDS survivor. On discharge, chest CT scan showed multiple ground-glass attenuation in both lungs, with traction bronchiectasis; at 3-month follow-up, chest CT scan showed reticular, ground-glass attenuation was diminished from discharge, and traction bronchiectasis was still visible. **C** Chest CT fibrosis score in COVID-19 ARDS patients from admission to 3 months post-discharge. **D, E** Pulmonary function tests at discharge and 3-month follow-up. ^*^p < 0.05, ^**^p < 0.01, ^***^p < 0.001. NS, no statistically significant difference

Thirty-seven patients in the COVID-19 ARDS group survived discharge, and they underwent chest CT and pulmonary function test on the day of discharge. Three months after discharge, all survivors of the COVID-19 ARDS group were followed up in the specific outpatient clinic. Normal chest CT at 3-month follow-up was present in 16/37 (43%) patients (Fig. 5A). Abnormalities on 3-month chest CT were found in 21/37 (57%) patients (Fig. 5B). Reticulation, ground-glass attenuation, and traction bronchiectasis were frequently CT signs associated with pulmonary fibrosis at follow-up. We also scored pulmonary fibrosis on the follow-up chest CT, and our data showed that the CT fibrosis scores at the 3-month follow-up were significantly lower (p < 0.001) than discharge (Fig. 5C). Correspondingly, lung function follow-up also showed significant improvement in forced vital capacity (FVC) % of predicted (p < 0.001) and diffusing capacity of the lung for carbonmonoxide (DLCO) % of predicted (p < 0.001) at 3 months compared to discharge (Fig. 5D, E).

**Fig. 6:**
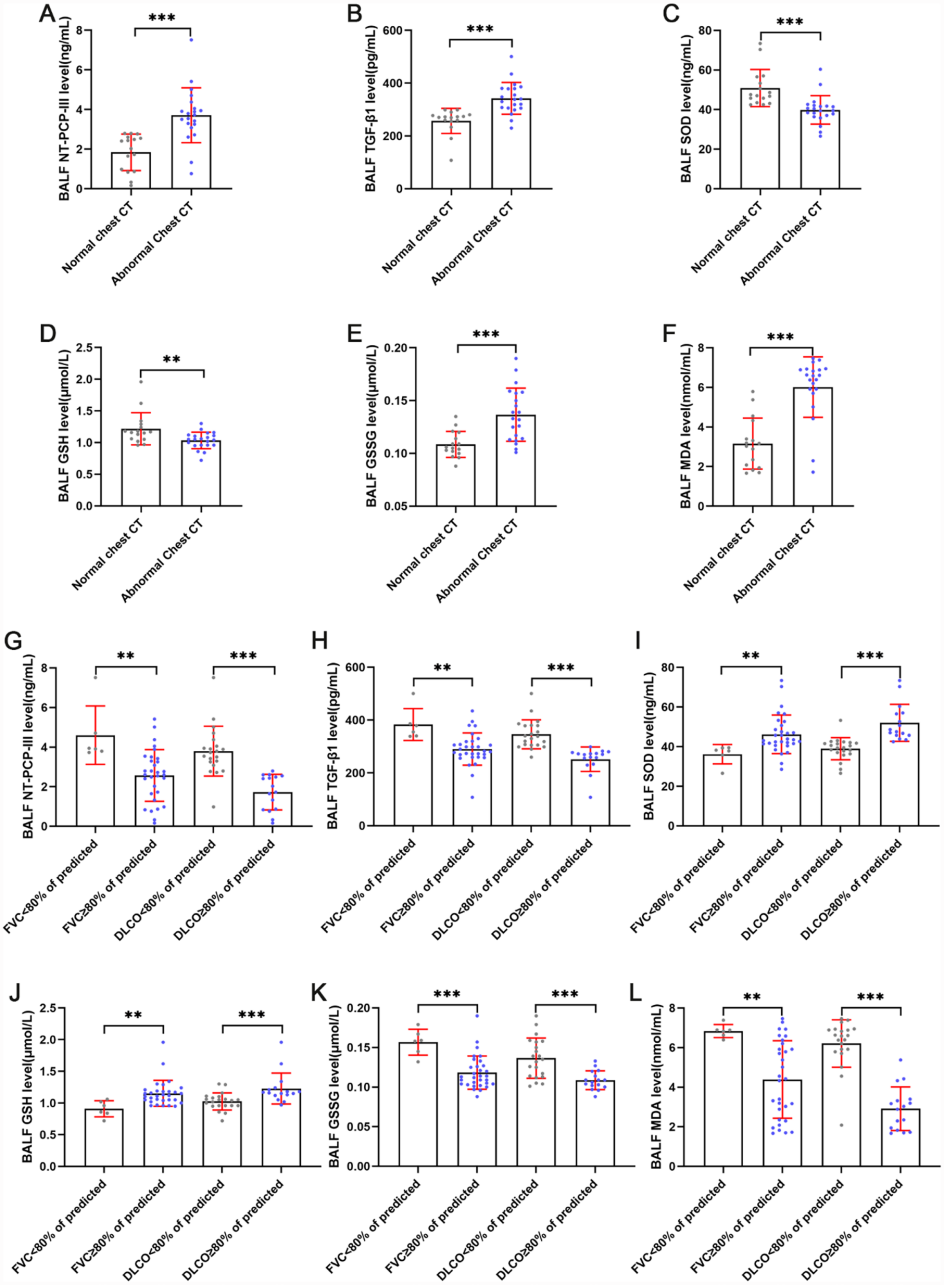
Association of lung abnormalities at 3-month follow-up with pulmonary fibroproliferation and redox imbalance in COVID-19 ARDS survivors. **A–F** Relationship between chest CT abnormalities at 3-month follow-up and NT-PCP-III, TGF-β1, SOD, GSH, GSSG, and MDA levels in bronchoalveolar lavage fluid on admission. **G–L** Relationship between lung ventilation abnormality (FVC < 80% of predicted) or lung diffusion capacity impairment (DLCO < 80% of predicted) at 3-month follow-up and NT-PCP-III, TGF-β1, SOD, GSH, GSSG and MDA levels of bronchoalveolar lavage fluid on admission. ^*^p < 0.05, ^**^p < 0.01, ^***^p < 0.001. NS, no statistically significant difference

COVID-19 ARDS survivors were divided into two groups based on follow-up chest CT manifestations: normal chest CT and abnormal chest CT. Not surprisingly, admission BALF pulmonary fibroproliferative biomarkers NT-PCP-III (p < 0.001) and TGF-β1 (p < 0.001) were significantly higher in the abnormal chest CT group than the normal chest CT group (Fig. 6A, B). Additionally, we observed decreased SOD (p < 0.001) and GSH (p < 0.01) levels, and increased GSSG (p < 0.001) and MDA levels (p < 0.001) in BALF on admission in the abnormal chest CT group compared to survivors in the normal chest CT group (Fig. 6C–F). These results imply that abnormalities at 3-month chest CT were associated with early pulmonary redox imbalance in COVID-19 ARDS. Lung ventilation abnormality (FVC < 80% of predicted) and lung diffusion capacity impairment (DLCO < 80% of predicted) were frequent in pulmonary function tests 3-month follow-up data. To investigate the factors that influence lung function abnormalities in survivors, we further compared fibroproliferative and redox status biomarkers in BALF at enrolment between survivors with normal and abnormal lung function (Fig. 6G–L). Of note, levels of NT-PCP-III (p < 0.01), TGF-β1 (p < 0.01), GSSG (p < 0.001), and MDA (p < 0.01) in admission BALF were elevated in survivors with FVC < 80% of predicted compared to survivors with FVC ≥ 80% of predicted, accompanied by decreased SOD (p < 0.01) and GSH (p < 0.01) levels. Showed similar trends, NT-PCP-III (p < 0.001), TGF-β1(p < 0.001), GSSG (p < 0.001), and MDA levels (p < 0.001) in admission BALF were higher in DLCO < 80% of predicted survivors than DLCO ≥ 80% of predicted survivors, whereas SOD (p < 0.001) and GSH (p < 0.001) levels were significantly lower. Overall, these data provide evidence that long-term residual pulmonary fibrosis and lung function impairment in COVID-19 ARDS survivors were associated with early pulmonary fibroproliferation and redox imbalance.

## Discussion

The present study demonstrates that pulmonary redox imbalance drives fibroproliferation at the early stages of moderate/severe COVID-19 ARDS. In particular, this study details the impact of pulmonary redox imbalance in COVID-19 ARDS patients on clinical outcomes, post-discharge residual pulmonary fibrosis, and lung function impairment.

Unlike SARS-CoV-1, which has not been endemic in the community since 2003, SARS-CoV-2 does not appear to be fading away [45]. Moreover, the newer SARS-CoV-2 variant has been shown to evade previously infected and vaccinated antibodies, resulting in new infections and re-infections worldwide [46]. Although COVID-19 is a systemic disease, the lung is the primary target of infection and injury, resulting in ARDS in severe cases [47]. We observed significantly higher peripheral blood ferritin and D-dimer concentrations in enrolled COVID-19 ARDS patients than in non-ARDS patients (Table 2), reflecting an impaired adaptive immune response and an underlying thromboinflammatory state [48, 49]. However, low oxygenation index (PaO_2_/FiO_2_) and mechanical ventilation dependence were the primary reasons for these enrolled COVID-19 ARDS patients to receive intensive care treatment (Table 2). The pathology of ARDS is usually considered to include three overlapping phases, in which an initial inflammatory exudative phase is followed by a fibroproliferative phase. During the fibroproliferative phase, fibroblasts and myofibroblasts migrate, replicate, and secrete extracellular matrix components such as collagen I and collagen III. Unabated, this process can ultimately lead to the establishment of pulmonary fibrosis [2]. In our cohort of COVID-19 ARDS patients (Fig. 2), the bilateral multiple ground-glass attenuations commonly observed on chest CT at admission likely represent the imaging-associated manifestation of diffuse alveolar injury, inflammatory cell infiltration, airspace edema, and interstitial fibroproliferation [50]. The gold standard for diagnosing pulmonary fibrosis remains lung biopsy and histopathology, but this is a risky operation [6]. In this study, NT-PCP-III, a biomarker of collagen synthesis, was used to assess fibroproliferation prior to the establishment of pulmonary fibrosis [51]. Notably, we observed that NT-PCP-III levels were significantly elevated in BALF from COVID-19 ARDS patients on admission compared to non-ARDS patients (Fig. 2). In addition, TGF-β1 was detected in BALF at a consistent trend with NT-PCP-III suggesting an early upregulation of the pro-fibrotic pathway [52]. The present data indicated that the pulmonary fibroproliferative response occurs early in COVID-19 ARDS, suggesting that prevention of pulmonary fibrosis should begin at the time of ARDS diagnosis.

In the pathophysiology process of SARS-CoV-2 infection, redox imbalance is both a cause and a consequence, ultimately leading to oxidative stress, oxidative damage at the epithelial-endothelial interface, and associated cell degeneration [53, 54]. Previous studies observed the redox status by detecting tissue GSH and GSSG concentrations, the antioxidant capacity by detecting SOD activity, and the level of oxidative damage by detecting MDA concentration [55, 56]. Given our concern for redox homeostasis in the lungs, we examined the BALF of all enrolled patients on admission. The present results showed that the oxidative damage biomarker (MDA) level was increased and the antioxidant capacity (SOD and GSH) was significantly decreased in the COVID-19 ARDS group compared with the non-ARDS group (Fig. 3). This is consistent with the results of quantitative proteomic analysis in BALF from critical COVID-19 patients by Hao-Long Zeng et al. [57]. All these results provide evidence that pulmonary redox balance is disrupted and AECs are in a state of oxidative stress in COVID-19 ARDS. In vivo research has demonstrated that pulmonary redox imbalance plays an important role in the pathophysiological mechanisms of pulmonary fibrosis after acute lung injury [58]. However, the relationship between pulmonary redox homeostasis and pulmonary fibrosis has not been investigated in cases of severe COVID-19 or ARDS. Our spearman correlation analysis shows a direct correlation between oxidative damage biomarkers in BALF and CT scores of pulmonary fibrosis as well as fibroproliferative markers in BALF. Conversely, our analysis results also suggest an inverse relationship between antioxidants in BALF and CT scores of pulmonary fibrosis as well as fibroproliferative biomarkers in BALF (Fig. 3). The present data corroborate with previous in vivo findings that pulmonary redox imbalance triggers oxidative stress, which induces oxidative damage and drives a fibroproliferative response [59]. Furthermore, circulating redox imbalance has also been reported to correlate with COVID-19 severity [60, 61]. According to our subgroup analyses of survivors and non-survivors from the COVID-19 ARDS group, non-survivors showed more significantly increased levels of pulmonary fibroproliferation and redox imbalance compared to survivors. Such result suggests that pulmonary fibroproliferation and redox imbalance potentially increase the risk of death in patients with COVID-19 ARDS. Of interest, there was no significant difference in chest CT fibrosis scores between survivors and non-survivors on admission in our study, which is different from the result of the previous study [44]. Our enrolled patients were transferred from the emergency ward and were generally in the early stages of ARDS. Whereas chest CT scans were used to visualize established pulmonary fibrosis, the sensitivity and specificity for identifying the early fibroproliferative stages were poor compared with NT-PCP-III [6]. Besides, pulmonary condensation is an important limiting factor in the observation of pulmonary fibroproliferation and fibrosis scores [7].

In the fourth year of the SARS-CoV-2 pandemic, considerable progress has been made in understanding the long-term effects of COVID-19 on the lungs. Earlier studies have reported that a subset of COVID-19 survivors suffer from long-term residual pulmonary fibrosis and lung function impairment, which is consistent with our results of 3-month follow-up (Fig. 5) [14]. COVID-19 patients with fatigue, dyspnea, and cognitive impairment persisting for 3 months are referred to as long COVID, which has been demonstrated associated with residual pulmonary fibrosis and lung function impairment [62, 63]. Our results also showed significant absorption of residual pulmonary fibrosis and lung function improvement in COVID-19 survivors at 3-month follow-up compared with discharge (Fig. 5). Indeed, this absorption of residual pulmonary fibrosis challenges our previous understanding that established pulmonary fibrosis is irreversible [64]. The more convincing explanation at present is that pulmonary neovascularization plays a beneficial effect in abnormal lung recovery in some patients [31, 47]. Recent studies have reported a potential role for peripheral circulating redox imbalance in the pathogenesis of long COVID, yet the relationship between pulmonary redox imbalance and long-term lung abnormalities in COVID-19 survivors has rarely been reported [65, 66]. A common sequela of COVID-19 is pulmonary fibrosis [67]. The present data also indicate that residual pulmonary fibrosis and lung function impairment in COVID-19 ARDS survivors are associated with the early extent of pulmonary fibroproliferation (Fig. 6). Basic research has demonstrated that oxidative stress triggered by redox imbalance is involved in pulmonary fibrosis by stimulating fibroblast activation, fibroblast to myofibroblast differentiation, and increased ECM deposition [68]. Our investigations validated in the population that redox imbalance causes residual pulmonary fibrosis in COVID-19 ARDS survivors and results in impairment of lung ventilation and diffusion function by driving early pulmonary fibroproliferation. These results above indicate that pulmonary redox imbalance triggered oxidative stress plays an important role in the pathophysiology of COVID-19 as well as long COVID.

Previous studies have identified hyper-inflammatory responses as a main driver of severe COVID-19 and long COVID, and our study results add that pulmonary redox imbalance may also be a major mechanism [69]. The potential therapeutic value of antioxidants in COVID-19-related diseases has attracted the attention of an increasing number of researchers [70, 71]. Although there are still significant challenges in translating the current results into clinical practice, our findings might promote the inclusion of more antioxidants in randomized controlled trials. We believe that perhaps the future clinical management of ARDS patients might be more sensitive to the maintenance of pulmonary redox homeostasis and early prevention of pulmonary fibrosis.

Our study also has some limitations. First, it was a single-center study, and there may be some bias. Second, due to the long study period, we could not determine whether the SARS-CoV-2 variant affected our results. Other studies have found that sex, age, and duration of mechanical ventilation are associated with pulmonary fibrosis, whereas oxidative stress may be both a cause and a consequence of these risk factors.

## Conclusions

This study demonstrates that altering pulmonary redox balance drives an early pulmonary fibroproliferative response in COVID-19-associated ARDS. Pulmonary redox imbalance and fibroproliferation increase the risk of death in severe COVID-19 and affect the resolution of residual pulmonary fibrosis and recovery from lung function impairment post-COVID-19. Despite advances in using antioxidants and redox modulators to prevent and treat COVID-19 complications, this study provides a solid basis for testing compounds targeted at restoring pulmonary redox balance against COVID-19-associated pulmonary fibrosis.

## Data Availability

Data will be made accessible upon request.

## Abbreviations

COVID-19: Coronavirus disease 2019
ARDS: Acute respiratory distress syndrome
CT: Computed tomography
NT-PCP-III: N-terminal peptide of alveolar collagen III
TGF-β1: Transforming growth factor-beta1
ROS: Reactive oxygen species
RNS: Reactive nitrogen species
SOD: Superoxide dismutase
GSH: Reduced glutathione
GSSG: Oxidized glutathione
MDA: Malondialdehyde
SARS-CoV-2: Severe acute respiratory syndrome coronavirus 2
RNA: Ribonucleic acid
BAL: Bronchoalveolar lavage
BALF: Bronchoalveolar lavage fluid
ALI: Acute lung injury
AECs: Alveolar epithelial cells
EMT: Epithelial-mesenchymal transition
ECM: Extracellular matrix
NRF2: Nuclear factor (erythroid-derived 2)-like 2
ELISA: Enzyme-linked immunosorbent assay
BMI: Body mass index
APACHE II: Acute physiology and chronic health evaluation II
SOFA: Sequential organ failure assessment
PaO_2_/FiO_2_: Arterial oxygen tension pressure/fraction of inspired oxygen; oxygenation index
CRP: C-reactive protein
FVC: Forced vital capacity
DLCO: Diffusing capacity of the lung for carbonmonoxide
